# End-to-end SARS-CoV-2 transmission risks in sport: Current evidence and practical recommendations

**DOI:** 10.17159/2078-516X/2021/v33i1a11210

**Published:** 2021-01-15

**Authors:** B Jones, G Phillips, F Valeriani, T Edwards, ER Adams, L Bonadonna, RJ Copeland, MJ Cross, C Dalton, L Hodgson, A Jimenez, SP Kemp, J Patricios, V Romano Spica, KA Stokes, M Weed, C Beggs

**Affiliations:** 1 Carnegie Applied Rugby Research (CARR) centre, Carnegie School of Sport, Leeds Beckett University, Leeds, UK; 2 England Performance Unit, The Rugby Football League, Leeds, UK; 3 Leeds Rhinos Rugby League club, Leeds, UK; 4 Division of Exercise Science and Sports Medicine, Department of Human Biology, Faculty of Health Sciences, the University of Cape Town and the Sports Science Institute of South Africa, Cape Town, South Africa; 5 School of Science and Technology, University of New England, Armidale, NSW, Australia; 6 Hull Kingston Rovers, Hull, UK; 7 Public Health Unit, Department of Movement, Human and Health Sciences; University of Rome “Foro Italico”, Rome, Italy; 8 Department of Tropical Disease Biology, Liverpool School of Tropical Medicine, Pembroke Place, Liverpool, L3 5QA, UK; 9 Italian National Institute of Health, Rome Italy; 10 Advanced Wellbeing Research Centre, Sheffield Hallam University, UK; 11 University of Bath, Bath, UK; 12 Premiership Rugby, Twickenham, UK; 13 The Football Association, St George’s Park, Burton-Upon-Trent, UK; 14 School of Clinical and Applied Sciences, Leeds Beckett University, Leeds, UK; 15 Centre for Sport Studies, King Juan Carlos University, Fuenlabrada, Madrid, Spain; 16 Rugby Football Union, Twickenham, UK; 17 London School of Hygiene and Tropical Medicine, London, UK; 18 Wits Sport and Health (WiSH), School of Clinical Medicine, Faculty of Health Sciences, University of the Witwatersrand, Johannesburg, South Africa; 19 Centre for Sport, Physical Education and Activity Research (spear), Canterbury Christ Church University, UK

**Keywords:** COVID-19, virus, illness, infection

## Abstract

The coronavirus disease 2019 (COVID-19) pandemic has caused disruption to professional and recreational sports across the world. The SARS-CoV-2 virus can be transmitted by relatively large respiratory droplets that behave ballistically, and exhaled aerosol droplets, which potentially pose a greater risk. This review provides a summary of end-to-end SARS-CoV-2 transmission risk factors for sport and an overview of transmission mechanisms to be considered by all stakeholders. The risk of SARS-CoV-2 transmission is greatest indoors, and primarily influenced by the ventilation of the environment and the close proximity of individuals. The SARS-CoV-2 transmission risks outdoors, e.g. via water, and from fomites, appear less than initially thought. Mitigation strategies include good end-to-end scenario planning of activities to optimise physical distancing, face mask wearing and hygiene practice of individuals, the environment and equipment. The identification and removal of infectious individuals should be undertaken by means of the taking of temperature and COVID-19 symptom screening, and the use of diagnostic monitoring tests to identify asymptomatic individuals. Using adequate video footage, data from proximity technology and subject interviews, the identification and isolation of ‘close contacts’ should also be undertaken to limit SARS-CoV-2 transmission within sporting environments and into the wider community. Sports should aim to undertake activities outdoors where possible, given the lower SARS-CoV-2 transmission risk, in comparison to indoor environments.

The coronavirus disease 2019 (COVID-19) pandemic has caused disruption to professional and recreational sports across the world.^[1]^ The causal agent of COVID-19 is the Severe Acute Respiratory Syndrome Coronavirus 2 (SARS-CoV-2)^[2]^, which is transmitted from human-to-human by multiple pathways.^[3,4]^ The novelty of the SARS-CoV-2 virus and therefore population susceptibility, in addition to the high transmissibility of the SARS-CoV-2 virus, ultimately resulted in the COVID-19 pandemic.^[5]^

Professional and recreational sports are obligated to implement risk management and mitigation strategies to prevent SARS-CoV-2 transmission and the subsequent COVID-19 spread.^[6,7]^ The SARS-CoV-2 virus can be transmitted by three main routes in sport; respiratory aerosol, droplets and fomites.^[8,9]^ The risks of SARS-CoV-2 transmission are influenced by the environment in which they occur (Fig. 1). All activities, from leaving home, including time spent within the sporting environment, and other associated activities (e.g. meetings and travel) should be considered as SARS-CoV-2 transmission risks (Fig. 2).^[10,11]^

The purpose of this review is to provide an overview of the considerations of end-to-end SARS-CoV-2 transmission risk and practical steps for risk management within sport.

## SARS-CoV-2 transmission via respiratory aerosol and droplets

The SARS-CoV-2 virus can be transmitted by larger respiratory droplets (>100 μm in diameter) that behave ballistically, and smaller exhaled aerosol droplets (<100 μm in diameter), which potentially pose a greater risk.^[12–14]^ This is because aerosol droplets rapidly evaporate to become small aerosol particles (<50 μm in diameter)^[15,16]^ that can easily be inhaled.^[13,17]^ Furthermore, smaller aerosol droplets can travel much further than ballistic droplets on convection air currents. These smaller aerosol droplets pose both a ‘near-field’ and a ‘far-field’ threat. Near-field refers to close proximity (e.g. 1–2 m), and far-field is beyond 2 m. Near-field SARS-CoV-2 transmission risks are caused by clouds of exhaled aerosol particles, potentially infecting individuals within close proximity. Far-field SARS-CoV-2 transmission risks occur primarily indoors when aerosol particles have been dispersed by air currents into the wider room space. The near-field transmission risks posed by large respiratory aerosol droplets can be mitigated by physical (or social) distancing, protective screens, and the use of face masks.^[18,19]^ The far-field risks posed by smaller infectious aerosols must be countered either by ventilation which flushes airborne particles from the room space,^[19]^ or potentially by air disinfection which biologically inactivates the SARS-CoV-2 virus.^[20]^ In addition, thermal plumes, warm convection air currents that surround all individuals, appear to play an important role in the transportation of respiratory aerosols,^[21]^ with exhaled fine aerosol particles rising vertically above the heads of individuals. Indoors, the aerosol particles entrained into thermal plumes become trapped by the ceiling and so get recirculated around the room space on convection currents and as such become a SARS-CoV-2 transmission risk within far-field zones.

The transmission risk of the SARS-CoV-2 virus is far greater indoors than outdoors, primarily due to increased ventilation outdoors. Although near-field SARS-CoV-2 transmission can occur both indoors and outdoors,^[22]^ in the outdoor environment the near-field transmission risk is lower because air velocities are generally higher, with the result that the exhaled aerosol particles will be dispersed more rapidly. This dispersion (dilution) effect becomes less pronounced the closer individuals are to each other. The risk of far-field aerosol transmission is much greater indoors because the concentration of aerosol particles in room spaces builds up over time, particularly in poorly ventilated spaces. The risk is further increased by aerosol particles, which are entrained in thermal plumes, trapped by the ceiling, before slowly descending through the breathing zone due to gravitational deposition. Outdoors, these are dispersed upward into the atmosphere, and therefore far-field aerosol transmission is unlikely outdoors given greater ventilation.^[23]^

To determine the SARS-CoV-2 transmission risk, it is important to define indoor and outdoor environments. This can be challenging in sport due to covered outdoor spaces (e.g. ‘indoor’ stadiums). Therefore, evaluating spaces based on near-field and far-field aerosol threat may be more appropriate. In a poorly ventilated communal changing area, both the near-field and far-field threats will be significant, whereas in a large indoor training facility with no ceiling and a high roof space, the far-field aerosol threat will be much less. This is due to the greater volume of the space, thus reduced concentration of SARS-CoV-2 viral particles potentially inhaled over time. If air movement at low level is poor (i.e. low air velocities) then the near-field threat may still be high, therefore physical distancing and mask wearing could be beneficial. Many larger indoor sporting facilities (i.e. arenas) normally contain audiences, in which case, because of the high numbers of people involved, this may result in air and ventilation characteristics behaving similar to an indoor space for COVID-19 risk assessment purposes.

The risk posed by far-field aerosol transmission of the SARS-CoV-2 virus can be mitigated through improved room ventilation (e.g. opening windows and doors).^[24,25]^ It has been shown that the SARS-CoV-2 virus survives in aerosols for longer when the air is cooler and drier.^[21,26]^ The viral half-life is >10 times longer outdoors during the winter and autumn months compared with the summer.^[25]^ Even though a room space may be heated in winter, the air can still be very dry (e.g. 10–40% relative humidity). The viral load in any droplets inhaled may be substantially greater than would be the case during the summer months.^[25]^ While the implications of this are not yet fully understood, it may explain in part the seasonal variation in COVID-19 case numbers that have been observed in many temperate regions. UVB radiation in sunlight has also been shown to rapidly degrade the virus. Consequently, COVID-19 appears to have a seasonal component,^[27]^ with transmission greatly reduced during the summer months when temperatures and UVB levels are higher and the air is more humid.

The probability that far-field transmission events will occur can be determined from the Wells-Riley equation (Equation 1).^[28]^ The probability of acquiring an infection by the airborne route increases as: (i) the number of infectious people present increases; (ii) the quanta generation rate increases; and (iii) the number of people susceptible spend longer in the presence of infectious people. The quanta generation rate, *q*, cannot be obtained directly, but rather, must be estimated epidemiologically from outbreak data. With respect to this, a quantum of infection is defined as the infectious dose required to infect 63.2% of the susceptible people present.^[28]^


Equation 1 $$C=S\left(1-e^{\left(-\frac{Iqpt}{Q}\right)}\right)$$

Where: *C* is the number of new infection cases; *S* is the number of susceptible individuals; *I* is the number of infectors; *p* is the average pulmonary ventilation rate of a person (m^3^/s); *q* is the quanta generation rate of the infectious agent (quanta/s); *t* is the exposure time (s), and *Q* is the room ventilation rate with clean outside air (m^3^/s).

To reduce the SARS-CoV-2 transmission risk by the aerosol route in any given context, it is important to minimise the number of people present and the duration of exposure, and maximise the room ventilation rates so that the concentration of infectious particles in the air is reduced.^[29–30]^ It is not always easy to determine room ventilation rates, particularly in situations where natural ventilation is employed. Therefore, monitoring carbon dioxide (CO_2_) can be used as a surrogate measure for ventilation in the Wells-Riley model.^[31]^

## SARS-CoV-2 transmission via fomites

Fomites may contribute to the transmission of SARS-CoV-2. High viral loads of SARS-CoV-2 have been shown to remain viable for up to 72 hours on inert surfaces, whilst undergoing exponential decay.^[32]^ Viral decay in culture media is increased at higher temperatures, with the virus remaining infectious for seven days at 22°C, one day at 37°C and 30 minutes at 56°C.^[33]^ Temperature is therefore likely to be a key determinant of surface stability. As equipment is commonly shared in numerous sports, this does potentially pose a route of transmission,^[34,35]^ although the likelihood of transferring a sufficient amount of the virus to cause an infection to the mucus membranes of another person remains unclear.^[34]^ Viral shedding into the environment has been demonstrated during SARS-CoV-2 infection. The rooms of hospitalised SARS-CoV-2 patients can be heavily contaminated with SARS-CoV-2 RNA, including frequently touched surfaces, such as sinks and door handles.^[36]^ The transfer of SARS-CoV-2 RNA from the hands of patients to objects has also been documented, demonstrating the environmental contamination originating from infectious individuals.^[37]^ However, most studies have utilised quantitative reverse transcription polymerase chain reaction (RT-qPCR) to detect SARS-CoV-2 ribonucleic acid (RNA) in the environment, rather than demonstrating infectious viral particles using culture. A study of environmental SARS-CoV-2 in the rooms of quarantining confirmed positive cases found 29/55 surfaces in the rooms of symptomatic cases were positive for SARS-CoV-2 by RT-qPCR; however, no viable virus was isolated in cell culture.^[38]^ As such, the contribution of fomites to the transmission of SARS-CoV-2 is controversial,^[39]^ and remains relatively unknown,^[25]^ in part due to no minimum infectious dose of SARS-CoV-2 being established,^[40]^ hampering studies of transmission dynamics from surfaces.

The risk of SARS-CoV-2 transmission via saliva, due to the potential to carry high viral loads,^[41]^ is significant. The risk is greater in sports where spitting is common practice (e.g. in cricket, saliva is often used to shine cricket balls, potentially facilitating the deposition of viral particles from infected players). RNA from an inactivated SARS-CoV-2 virus can be detected from the surface of cricket balls up to one hour post-inoculation using RT-qPCR, although viral viability (i.e. the ability to cause infection) cannot be determined using this approach.^[35]^ Using live SARS-CoV-2 viruses, an exponential reduction in detectable SARS-CoV-2 virions for all inoculated sport equipment (i.e. cricket glove, football, golf ball, horse saddle, rugby ball, tennis ball, gym pit foam) was observed over a short-term period (one minute to 90 minutes).^[34]^ The low inoculum (5.4×10^2^ virions, representing a 40μl saliva droplet from a SARS-CoV-2 infected player with a viral load in the lower quartile of cases) was only detectable on one (the polyurethane horse racing saddle) of ten materials at five minutes and no virus could be detected on any material after 15 minutes. These findings suggest that from individuals with lower viral loads, there is probably insufficient viral load transferred from fomites to be infectious.

The material composition should also be considered when evaluating the risk of SARS-CoV-2 transfer on sports equipment. For example, Edwards et al.^[34]^ found that viral recovery was reduced by absorbent materials, which included leather (e.g. red cricket ball and cricket glove) and polyurethane foam (e.g. gym mat foam). Harbourt et al.^[42]^ also demonstrated that viral stability was reduced on absorbent clothing in comparison to skin or plastic materials. The observation that porous materials result in reduced viral recovery and transmission risk can be used to prioritise materials for within-game cleaning or swapping, and focus cleaning efforts to reduce their effect on sporting events.^[34]^ In addition to the material composition of the sports equipment, the specific finish also appears important. For example, considering two bovine leather cricket balls, the ball that had synthetic grease on it had a lower viral recovery than the ball that had a nitrocellulose finish.^[34]^ There is potential for this information to be used by developers of sporting materials to engineer products to be less amenable to viral transmission.

The quantification of the viral load that may be transferred from an individual with a SARS-CoV-2 infection onto sports equipment has not been evaluated. Edwards et al.^[34]^ used previously reported concentrations seen in respiratory tract secretions, although the concentrations may be different in practice. A quantitative microbial assessment of the risk of infection from fomites has been performed using the Monte Carlo simulation.^[43]^ A lower than 1/10 000 infection risk was observed from a single touch of surfaces infected with a range of 1 to 10 000 genome copies/cm^2^. This supports the potentially limited role of fomites in SARS-CoV-2 transmission, although further research is still required.

## SARS-CoV-2 transmission via water

The transmission risk of the SARS-CoV-2 virus in water is an important consideration during the COVID-19 pandemic for sports. SARS-CoV-2 is an enveloped virus, similar to Orthomyxoviridae (e.g. influenza viruses), Paramyxoviridae (e.g. measles virus, mumps virus, respiratory syncytial virus), Herpesviridae, Coronaviridae (some with low pathogenicity, others with high pathogenicity like SARS-CoV and MERS-CoV). Whilst influenza viruses and coronaviruses can be found in trace amounts in faecal material and aqueous environments, waterborne infections have not been recorded.^[44–46]^ Coronaviruses may be introduced into aquatic habitats through urban or agricultural runoff or via wastewater effluents, as observed in lake, river and coastal waters.^[47–49]^ These viral units are likely to experience considerable decay and loss of infectivity rapidly after arriving in water.^[50]^

Several factors can influence virus survival, vitality and infectious capability in water, which include temperature, presence of suspended solid and organic matter, pH, and water treatments and disinfections.^[51]^ RNA fragments of the SARS-CoV-2 virus have not been detected in treated waters,^[52]^ therefore, current water treatment practices are likely to be effective in virus removal. Therefore, water is not considered a major transmission risk of SARS-CoV-2 for sports, due to the instability of water^[53]^ and susceptibility to oxidants, such as chlorine.^[54]^

## Transmission risk management considerations for sporting activities

**Fig. 2:**
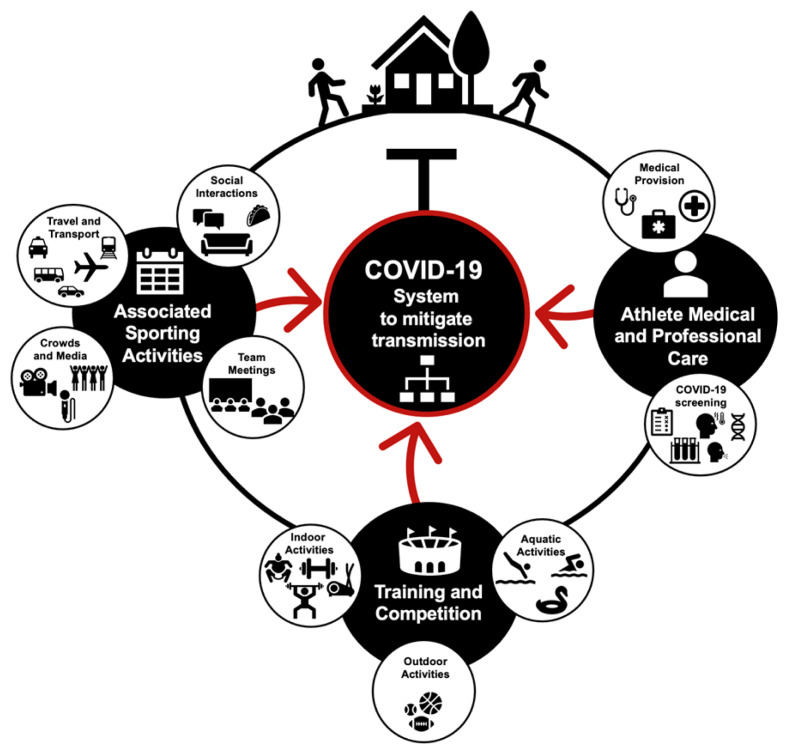
End-to-end transmission risk activities for sports

Fig. 2 presents activities that should be considered as end-to-end SARS-CoV-2 transmission risks within sport. A wider COVID-19 management system, including the identification, and removal of infectious and potentially infectious (e.g. individuals exposed to infectious individuals) should also form part of the overall SARS-CoV-2 risk mitigation strategy.

## Athlete medical and professional care

### Screening and testing for COVID-19

The risk of the SARS-CoV-2 spreading within a sporting environment can be reduced by the implementation of appropriate COVID-19 protocols, which aim to identify and prevent symptomatic and asymptomatic individuals entering the environment. The primary symptoms (e.g. loss of taste or smell, new or continuous cough, high temperature [>37.8°C], muscle aches and fatigue,^[55]^) can be monitored daily, and integrated within routine wellbeing types which are common in sport.^[56]^ The effectiveness of temperature screening with non-contact thermometers, in isolation of other monitoring strategies, has recently been questioned.^[57]^ Once symptomatic individuals are identified, they can then receive a consultation with a clinician, and/or be referred for appropriate SARS-CoV-2 diagnostic testing.

Asymptomatic and pre-symptomatic individuals with COVID-19 can have high viral loads, similar to those with clinical disease, indicating that athletes without symptoms are able to transmit the SARS-CoV-2 virus within a sporting environment.^[58]^ Various SARS-CoV-2 diagnostic testing protocols exist to identify symptomatic and pre-symptomatic individuals. The implementation of a testing programme would likely be influenced by both clinical reasoning, cost and logistical considerations.

The most sensitive tests for acute SARS-CoV-2 infection are RT-qPCR assays, which amplify and detect specific sequences of the SARS-CoV-2 genome. Multiple commercial and approved (Food and Drug Administration [FDA], Conformitè Europëenne in vitro diagnostic medical devices [CE-IVD]) assays are available to target either single or multiplex regions of the SARS-CoV-2 genome.^[59]^ Best performing assays can detect SARS-CoV-2 as low as 100 genome copies/mL.^[60]^ RT-qPCR testing requires complex infrastructure, undertaken in specialist laboratories. Cycle threshold (Ct) values provide a proxy measure of viral load in a RT-qPCR test, with values under 30 considered high and highly infectious, values between 30–40 low, and >40 negative.^[61–63]^ Whilst the lack of a more accurate comparator test or ‘gold standard’ makes the true accuracy of RT-qPCR difficult to ascertain,^[64]^ sensitivity is thought to range between 70% and 98%, depending on sample type, gene target, and kit manufacturer.^[65–66]^ The specificity of RT-qPCR is high, with large studies estimating it to lie between 97.4 and 99.1%.^[67]^

Whilst RT-qPCR provides a high level of sensitivity, the duration of time from swab to results (e.g. transit to, and processing within a laboratory) and the high commercial cost (approx. £100 GBP/$140 USD if purchased in United Kingdom (UK) or R830 ZAR/$60 USD if purchased in South Africa) means that other diagnostic tests may be preferable. Lateral flow tests (LFT) are an alternative valid, point-of-care diagnostic tool that can detect individuals with high viral loads, within approximately 15 minutes from swab to test outcome.^[68]^ The cost per LFT test is approximately £5 GBP if purchased in UK (approx. $7 USD) or approximately R208 ZAR if purchased in South Africa (approx. $15 USD), significantly less than a RT-qPCR test. In independent evaluations (World Health Organisation Emergency Use Listing; WHO-EUL) approved tests have >80% sensitivity rising to >95% sensitivity in individuals with high viral load (Ct <30) and >98% specificity, although this can vary according to manufacturer.^[69]^ Sampling technique is important, given when swabs are self-taken and read by non-professionals, the sensitivity may be as low as 40%.^[70]^ As such, the implementation of LFT within sports requires careful planning and appropriate training of any staff taking swabs.

LFT will not capture all positive cases (due to inability to detect low viral loads) but may be appropriate as part of an asymptomatic monitoring strategy. If one positive LFT is detected within a cohort during routine LFT asymptomatic monitoring, this can be used as a trigger for a surge in daily testing or for the RT-qPCR testing of individuals or whole squads. Furthermore, the sample collection to result duration for SARS-CoV-2 diagnostic tests may also be a consideration for sports. Even though RT-qPCR has a higher sensitivity than LFT, the longer sample collection to result duration (15 mins vs. approx. 24–36 hrs) may mean that asymptomatic infectious individuals inadvertently remain within the sporting environment if athletes are training daily.

Alternative assays exist, including real-time loop mediated isothermal amplification (LAMP) technology, where swab and saliva samples can be used with minimal processing and extensive laboratory facilities are not required. The typical time to run this diagnostic test is approximately 20 minutes, from sample collection to result.^[71]^ Tests undertaken using LAMP technology still require molecular expertise, are not truly point-of-care, and the cost reflects the personnel and equipment required to perform them. In the National Institute for Health Research (NIHR)-funded evaluation, LAMP testing was 79% sensitive on asymptomatic individuals and 100% specific, although it missed more than 50% of cases in a Manchester, UK pilot.^[71]^ There seems to be little, if any, increase in sensitivity from LFTs to these simplified molecular tools with no extraction, yet they are more complex and expensive to implement.

To avoid false positives results, it may be recommended that individuals who have previously tested positive for SARS-CoV-2 are removed from the testing programme for 90 days, due to residual RNA remnants following SARS-CoV-2 acute infection. Re-infection is unlikely in the 90 days following an infection,^[72]^ although the evidence still remains unclear.^[73]^ The rollout of vaccination programmes around the world presents a new challenge for the management of athletes.^[74]^ At present, it is unclear how athletes should be managed within routine diagnostic testing cycles once vaccinated for the SARS-CoV-2 virus. Most vaccination regimens are using two doses to first prime, and then boost immunity.^[75]^ A level of protection is gained from the first vaccination, with a trial of the Pfizer-BioNTech BNT162b2 mRNA COVID-19 vaccine showing 52% efficacy after the first dose.^[76]^ Breakout infections following full vaccination are less severe and likely to be less transmissible.^[77,78]^ There is speculation that vaccinated individuals may still asymptomatically carry SARS-CoV-2 and contribute to its transmission,^[79]^ although data are lacking in this area and will become available via surveillance studies of vaccinated populations.

When determining the appropriateness of a diagnostic test for a specific cohort, it is important to also consider the community prevalence of COVID-19.^[80]^ As the prevalence declines, the positive predictive value also declines exponentially. Even with a diagnostic test with high sensitivity and specificity, at low population prevalence the results may be false positives, as the positive predictive value becomes small.^[67]^ As such, diagnostic testing should be one part of the overall COVID-19 risk management measures.

## Providing safe medical and professional care

The near-field and far-field transmission risk of the SARS-CoV-2 virus must be carefully considered in the delivery of safe medical and professional care to athletes in sport. Athletes are supported by large multidisciplinary teams, consisting of doctors, physiotherapists, nutritionists, psychologists, and masseurs, among others. Determining what care can be safely delivered remotely through telemedicine is the most effective means of mitigating any transmission risk.^[81]^ However, certain situations can only be conducted ‘face-to-face’ and consequently this increases potential SARS-CoV-2 exposure between individuals at close proximity. Care deemed necessary and essential, such as supervised rehabilitation, medical examinations and procedures, must be permitted in a risk-mitigated manner.^[82,83]^ Delayed or compromised care could negatively impact an athlete’s wellbeing and sporting performance both in the short and longer term, which may result in prolonged recovery, disablement, and impact a future career opportunity.

Pre-scenario planning most effectively mitigates the SARS-CoV-2 transmission risk. Reducing the duration and frequency of interactions can be effective, as clinical environments tend to be specialised and not easily relocated to optimise environmental conditions. National healthcare guidance on personal protective equipment (PPE) has been interpreted for the sporting setting, taking into account the sport-specific nature, including delivery of pitch-side medical care (Table 1),^[84,85]^ with the recommendation that athlete patients also wear a face covering for the duration of review.^[82]^ Safe and effective use of PPE is founded on good training to ensure it is worn correctly and the risk of self-contamination is minimised during application and removal (donning and doffing). Importantly, for those not of an allied healthcare care profession, in the absence of appropriate training, high grade (i.e., Level 3) PPE is of no more protection than a simple face covering. All equipment, including PPE, should be single use where possible and, if reusable, subject to appropriate sanitisation protocols.

Verbal components of consultations should be conducted with physical distancing respected, breaching this only when essential for examinations and other procedures. Aerosol-generating procedures (Table 2) can be a significant source of virus transmission when conducted on infectious individuals, due to the aerosolisation of respiratory droplets.^[8]^ Consequently, these situations require a higher level of mitigation, through increasing the standard of PPE, and also the need for a dedicated area to limit the exposure to bystanders, which must be subject to appropriate decontamination after use.^[84]^

Establishing a sport-specific injury risk profile can help provide COVID-19 safe updates to emergency action plans and thus ensure adequate equipment is available to account for PPE availability and sanitisation protocols for equipment between uses. Emergency care poses the greatest challenge, due to the need for a rapid response and propensity for these scenarios to involve aerosol-generating procedures. This can include head injuries which carries the potential for airway compromise and cardiac arrest. Both airway intervention and chest compressions are deemed potential aerosol-generating procedures.^[86]^ Level 3 PPE can take some time to apply, which impacts on the ability for the medical response team to rapidly respond. Depending on resource availability and staff familiarity with donning and doffing, organisations may wish to have staff already prepared in Level 3 PPE for high-risk settings in order to prevent any unnecessary delay in delivering prompt emergency care.^[84,85]^ In circumstances where only Level 2 PPE is available, or there is a delay in donning Level 3 PPE, airway interventions beyond simple manoeuvres are not recommended. In cardiopulmonary resuscitation (CPR), chest compressions can be commenced in addition to the use of an automated external defibrillator, provided that a face covering is applied to the casualty which does not impede airflow (i.e. oxygen mask or light cloth). In youth sport, where cardiac arrest can more commonly be triggered by a respiratory cause,^[87]^ ventilation is crucial to survival. Medical teams should discuss how they wish to manage this situation, as a delay could severely impact clinical outcomes for the casualty. Outside of elite sports protocols, it may be decided that full CPR will be started in the absence of suitable PPE, at a risk to the responders. However, staff should not be put under undue pressure to compromise their own health and safety in the absence of adequate PPE.

## Training and competition

### Transmission risk during outdoor sporting activities

The transmission risk of SARS-CoV-2 during outdoor sporting activities can be determined based on the proximity, duration of close proximity and whether individuals are directly facing each other.^[7,88]^ These factors determine the risk of infectious respiratory aerosol and droplet particles transferred from human-to-human. Outdoor team sports, which include a large number of prolonged close interactions, or encounters between athletes outdoors (e.g. start or end of a race) pose a potential risk for human-to-human SARS-CoV-2 transmission. Whilst SARS-CoV-2 transmission during close proximity prolonged interactions outdoors is plausible, to date there has not been any confirmed transmission observed in sport (e.g. rugby league and soccer), despite infectious players inadvertently participating.^[10,89,90]^ During training and match play, it would be assumed that participants have an increased respiration rate due to the demands of exercise, and thus a substantial increase in aerosol expiration.^[91]^ Deep exhalation causes a 4- to 6-fold increase in aerosol particle concentration, and rapid inhalation increases aerosol particle concentration by a further 2- to 3-fold.^[92]^ Consequently, the physiological demands of training and match play increases the SARS-CoV-2 transmission risk in comparison to rest, given the increased rate and concentration of infectious particles being expired.

During most sporting activities outdoors, the environmental conditions will likely mitigate the risk of SARS-CoV-2 transmission via expired infectious particles. The very high ventilation rates experienced outdoors, together with higher air velocities, will disperse and dilute the infectious respiratory aerosol particles, thus reducing their concentration prior to inhalation. The closer the proximity between individuals and the longer the duration of the close proximity interactions, the greater the transmission risk of SARS-CoV-2, even when outdoors.^[7,93]^ Some outdoor sporting activities may create indoor-like environmental characteristics (e.g. low ventilation, poor air flow), and therefore may remain high risk for transmission.^[88]^ For example, a team huddle, face-to-face wrestling action, or rugby scrum may reduce ventilation, and therefore respiratory aerosol and droplet particles may behave in a similar way to indoor interactions, thus increasing the SARS-CoV-2 transmission risk. However, to date, no SARS-CoV-2 transmission has been reported during these activities.

The outdoor transmission risk appears to be lower than first suggested, which has since been reflected in the modification of outdoor contact-tracing frameworks in sport.^[7,88]^ Whilst the SARS-CoV-2 transmission risk during outdoor team sports appears to have been downgraded, the data available only provides a preliminary insight into the overall risk, given the small sample sizes within the respective studies.^[10,89,90]^ For example, in rugby league, eight infectious players inadvertently participated in matches with 100 other players.^[10]^ In the 14 days following the matches, five players tested positive for SARS-CoV-2 via RT-qPCR, although these positive cases were most likely traced to social interactions, car sharing and wider community transmission and were not linked to in-match transmission.

In outdoor evasion team sports, the overall purpose is to avoid the opposition. Thus in some sports, this will mean that during the match close proximity interactions between players are rare and may only be fleeting in nature.^[10]^ The greatest risk of SARS-CoV-2 transmission during outdoor sporting activities may be activities that are pre-match or proceed after that training activity, match or competition.^[94]^ Close proximity conversations, drink breaks, and pre-match, post-match or celebratory huddles may pose the greatest risk of SARS-CoV-2 transmission, and should therefore be considered within sports risk mitigation strategies for outdoor sport activities.

### Transmission risk during indoor sporting activities

In addition to the aforementioned outdoor risk factors (e.g. ventilation of space, duration of close proximity between individuals), further considerations in indoor training settings should be given to the increased breathing frequency and particle expiration, particularly with moderate to vigorous aerobic activities.^[91,92,95]^ Activities associated with forced exhalation and deeper breathing have been shown to generate aerosol droplets that travel beyond 2 m,^[96,97]^ extending the near-field transmission zone. The distance droplets and aerosols spread in the air can also be influenced by how an athlete moves within a space, given the risk of aerosol cloud formation within poorly ventilated spaces. Mitigation strategies to compensate this include increasing physical distancing beyond 2 m, positioning equipment so that people face away from each other, the use of screens, and avoiding loud background music (which requires individuals to shout to be heard, further increasing the generation of aerosols and droplets).

The regulations on the use of face coverings in indoor settings vary between countries, and use during exercise is an area of debate.^[98–101]^ It may provide some discomfort and sweating can result in the mask becoming damp, although the risk to the mask wearer appears minimal. Some indoor training activities may be more amenable for mask wearing (e.g. resistance training). Furthermore, if physical distancing is inadvertently and temporarily breached whilst moving around indoors, face coverings may provide a further mitigation strategy to reduce the risk of SARS-CoV-2 transmission. This may also help mitigate inadvertent high-risk SARS-CoV-2 transmission situations (e.g. where people may congregate; entrances and exits, changing rooms and lockers, holding areas). Physical distancing, the wearing of face coverings by staff and athletes where possible, and avoiding talking to others during or immediately after exercise can help reduce the SARS-CoV2 transmission risk during indoor sporting activities.

Fomite transmission should also be considered in relation to all surfaces, prioritising high-contact areas (e.g. drinking facilities, shared equipment, clothing). Sweat does not appear to be a transmission mechanism for SARS-CoV-2,^[102]^ thus surface contamination would be via fomites, caused by infectious respiratory aerosol and droplets. Individual labelled drinks containers, clothing and towels, in addition to appropriate cleaning practices (e.g. paper towel, disinfectant spray and bins close to equipment, and clothing washed at a temperature of 60°C or above immediately after use) can help mitigate the SARS-CoV-2 transmission risk.

### Transmission risk during aquatic activities

The potential transmission risk of SARS-CoV-2 during aquatic activities is via transmission in water and expired aerosol and droplet transmission, linked to the proximity (and potential overcrowding) between individuals. The poor resistance of enveloped viruses, such as SARS-CoV-2, to disinfected waters explains the limited transmissibility of this virus in water, regardless of the initial viral load.^[103–105]^ Furthermore, warmer water temperatures (i.e. >20–30° C) inactivate the virus quicker than lower temperatures (i.e., 4° C),^[106]^ and previous studies have suggested that some natural spa, waters may already have an intrinsic antibacterial activity due to their chemical and physical properties, as well as due to their resident microflora.^[107–109]^ The presence of viruses in the water of swimming pools is directly linked with contamination by bathers that could release traces of biological fluids, such as saliva or nasal mucus droplets, vomit or faeces.^[110,111]^ Pathogens and RNA fragments can be detected in recreational waters (e.g. swimming pools) for several reasons, including inadequate compliance with disinfection procedures and technical failures. However, the primary risk of SARS-CoV-2 transmission between individuals remains via respiratory aerosols and droplets.^[105,112]^

From a descriptive epidemiology perspective, the COVID-19 pandemic affected people involved in very different occupational, recreational, or physical activities, but no outbreaks have been associated to swimming pools. Physical distancing, masks and handwashing remain the key issues for prevention, and the practice of swimming itself would not represent a major risk compared to other activities and environments. Both swimming pools and spa waters do not seem to constitute a specific risk, at least according to current epidemiological data.^[105,112]^

The risk of SARS-CoV-2 transmission in water appears low, especially with the implementation of mitigation strategies. The SARS-CoV-2 transmission risk in aquatic sports appears linked to insufficient physical distancing whilst in swimming pools and spas, similar to other environments. Maintaining physical distancing, avoiding overcrowding by scheduling systems, and the implementation of a one-way system to avoid inadvertent clustering of individuals should be a priority. Similarly, optimising indoor ventilation and considering relative humidity and UV light (e.g. sunlight)^[20,24]^ will collectively reduce the transmissibility of SARS-CoV-2 within swimming pools and similar environments. Associated activities (e.g. changing rooms, travel) may pose the greatest SARS-CoV-2 transmission risk for aquatic sports. Unique to aquatic sports is the need for changing room access (in comparison to soccer for example), therefore these SARS-CoV-2 transmission risks should be considered, allowing similar risk mitigation strategies as other non-aquatic sports to be applied.

## Associated sporting activities

### Indoor meetings

Within sports, indoor individual and team meetings potentially pose a significant risk of SARS-CoV-2 transmission. Advice regarding SARS-CoV-2 transmission in educational settings is applicable to team meetings in sporting contexts. This can be broadly categorised as advice relating to near-field transmission (i.e. transmission via larger respiratory aerosol droplets and person-to-person contact), and far-field transmission (i.e. transmission via fine aerosol droplets that become truly airborne).

For any team meeting, it is advisable that individuals are seated at least 1 m (preferably 2 m) apart in an arrangement that avoids face-to-face exposure (i.e. participants seated behind each other rather than face-to-face). It is also advisable that face masks should be worn indoors, even when physical distancing is practised.^[18]^ This is not primarily to protect the wearers (although some limited protection appears to be afforded by wearing low-efficiency medical and cloth masks),^[113]^ but rather because there is evidence that wearing even low-quality masks reduces the emissions of SARS-CoV-2 virus-laden particles, both droplets and aerosols.^[114,115]^ Face masks block the shedding of ballistic droplets and larger aerosols and reduce the shedding of smaller respirable aerosols.^[114,116]^ While it can be argued that face coverings are not always necessary indoors when good physical distancing is exercised and spaces are well ventilated,^[117]^ given the wider implications associated with an athlete contracting COVID-19 (e.g. weakened teams, abandoned matches and tours), the wearing of face masks is recommended when attending meetings held indoors.

The airflow within a room is of paramount importance. It has been calculated that under steady-state conditions, the airborne viral load may reach as high as 1 248 RNA copies/m^3^ in a poorly ventilated room, simply due to the breathing by a super-emitter (Table 3).^[118]^ In order to mitigate airborne (far-field) transmission, it is necessary to ensure the room space is well ventilated with outside air (i.e. >10 L/s per person) to ensure CO_2_ levels are maintained below 1 000 ppm.^[19,31]^ If room CO_2_ levels exceed this threshold, strategies to increase the ventilation rate should be adopted. If the space is mechanically ventilated, the amount of outside air delivered should be maximised and the recirculated air minimised.^[19]^ Care should also be taken in buildings that employ centralised heating, ventilation and air conditioning (HVAC) systems. In such systems, to save energy during the winter months, up to about 80% of the extracted room air (return air) can be recirculated, with the result that aerosols containing the SARS-CoV-2 virus may be widely re-distributed around the building.^[19]^ If this is the case and it is not possible to convert the system to a full ‘fresh air’ system, then it may be necessary to retrofit ultraviolet (UVC) lamps, with a wavelength of 254 nm, into the return air ducts to disinfect the air and prevent recirculation of the virus.^[19]^

Whilst in team meetings, SARS-CoV-2 transmission risk can be worsened if the infector is talking loudly or shouting/singing. With an average sputum viral load, it has been estimated that speaking in a loud voice for one minute will generate >1 000 virion-containing aerosols.^[119]^ Therefore, theoretically a super-shedder, emitting a 100-fold higher viral load than average, could shed >100 000 virions in emitted droplets per minute of speaking.^[120]^ Applying findings from other settings, outbreaks have been reported in: nightclubs,^[14]^ religious gatherings,^[121,122]^ choirs and singing events,^[123]^ and weddings. All these settings involve a high density of individuals within confined spaces for considerable periods of time, with most involving singing or talking in order to be heard above the background noise.

While the short range (near-field) risk posed by ballistic droplets >100 μm can be mitigated by physical distancing, screens, and the use of face masks^[18,126]^ during team meetings, the longer range (far-field) threat posed by smaller infectious aerosols must be countered either by ventilation which flushes airborne particles from the room space, or by air disinfection which biologically inactivates the virus.^[20]^ The effectiveness of air purifiers can be determined via Clean Air Delivery Rate (CADR). The CADR indicates how many cubic meters of cleaned air the air purifier provides per hour and thus corresponds to the product of filter efficiency and volume flow rate that the unit circulates. At a CADR of 750 m^3^/h, the risk of infection per hour of time spent in a room with an infected person has been proposed to be reduced to 10%.^[127]^ Whilst the risk of infection is reduced, other mitigation strategies, such as ventilation and/or wearing masks, should also be implemented. Air purifiers can be used between meetings, which typically provide three to six air changes per hour, although higher air change values (e.g. 6) are recommended during the COVID-19 pandemic, to reduce the risk of infectious particles remaining within an environment.^[127]^ Further considerations should also be made when the air is cooler and drier, given that it has been shown that the SARS-CoV-2 virus survives in aerosols for considerably longer,^[27,28,128]^ and the viral half-life is >10 times longer during the winter and autumn months compared with the summer.^[25]^

### Breaks and social interactions

Similar to many other occupational settings, individuals in sport may have coffee and lunch breaks throughout the day. These breaks can become high SARS-CoV-2 transmission risk situations, due to multiple people congregating in one location (e.g. queuing in the canteen), touching the same object or surface (e.g. coffee machine), and people wanting to socialise and speak to each other whilst being less vigilant. This can be worsened by the fact that masks must be removed to eat and drink. It is therefore important to carefully consider how social breaks are managed, so as not to inadvertently undermine infection control measures taken elsewhere.

Given the above, it is important to plan both spatially and temporally how social breaks are undertaken. This is particularly important when team meetings are held in shared facilities that might be occupied by other groups (e.g. sports centres). This also applies to the serving and eating of food, whereby buffet style meal serving will pose a greater risk of SARS-CoV-2 than table service, due to the risk of clustering and human-to-human interactions. Where possible, people should be encouraged to physically distance themselves, eat and drink outdoors, and wear face masks up until the point where food or drink is consumed. It may also be advantageous to stagger meal timings to avoid large groups being within shared spaces without masks for a period of time. If consuming food and drink outdoors is not possible, then the seating and tables should be arranged to facilitate physical distancing in a well-ventilated space.

### Travel and transportation

SARS-CoV-2 transmission during travel poses a risk for elite sport, given that teams often travel in large groups both nationally and internationally. When planning travel arrangements, it is important to consider the mode of transport, required stops, and accommodation. In all situations, near-field risks can be mitigated via physically distancing, seating positions (e.g. seated behind, or side-by-side, but not facing other travellers ^[129]^), wearing face masks and minimising the unnecessary movement of individuals. During travel it is preferable that a distance of 2 m is maintained during the entire journey. This may also serve to reduce the density of people within a space ^[130]^ and also prohibit car sharing, due to the inability to maintain a physical distance of 2 m between individuals.

When making travel plans, it is important to consider the prevalence of COVID-19 in the destination population, as this will influence the likelihood of an interaction with an infectious native individual.^[80]^ If the prevalence is high, extra precautions may be required, including bubbling the athletic group, as there will be a much greater chance of interactions occurring with hotel staff, officials and other support or service staff. This group may also benefit from routine screening protocols, which may include symptom monitoring, and daily SARS-CoV-2 diagnostic testing.

Travel on commercial public transport vehicles introduces a higher level of SARS-CoV-2 transmission risk exposure than travelling on a privately chartered vehicle, due to potential interactions with unmonitored individuals. Chartered transport may not always be possible due to financial constraints. Therefore, it is advisable to keep records of designated named seating plans. During the journey, movement around and conversation should be avoided with travellers.^[129]^ The wearing of face masks will afford some protection to the wearer and prevent the dispersion of large respiratory droplets that could impact other travellers.^[116]^ Limiting unnecessary travel breaks, and time spent at potential ‘infection hubs’ (e.g. shared spaces within airports, such as passport control and security screening, train stations, and motorway service stations) is advisable throughout a journey. This ensures that the group interacts with fewer external environments and individuals. Journeys should be kept as short as possible in order to minimise overall risk to all involved.

The far-field transmission risk is dictated largely by the ventilation characteristics of the particular passenger vehicle in question. In cars and older style buses and trains, it is possible to open the windows to promote ventilation. Newer buses (coaches) and train carriages are typically hermetically sealed, and tend to overheat, especially in direct sunlight. As such they require mechanical cooling (air conditioning) and ventilation. This, however, consumes a considerable amount of energy,^[131]^ with the result that manufacturers and operators tend to recirculate most of the air and minimise the amount of ‘fresh’ outdoor air that is supplied to the carriages.^[132]^ CO_2_ concentrations >1 800 ppm^[132–134]^ and as high as 5 525 ppm^[135]^ have been recorded on urban railway carriages during peak periods (CO_2_ concentrations in well-ventilated spaces are generally <1 000 ppm).^[19,31]^ This suggests that during periods of high occupancy, ventilation rates in passenger carriages are frequently inadequate to protect passengers from far-field SARS-CoV-2 transmission. Given this, it is advisable for athletes to travel in a significantly reduced occupancy, which will also be a by-product of physical distancing measures.

Unlike trains and coaches, the air conditioning systems in aircraft cabins are generally fitted with high-efficiency particulate air (HEPA) filters which are highly effective at removing viral particles from recirculated air,^[11]^ keeping the supply clean and free of pathogens. They also allow a high air change rate to be maintained in the cabin space, which is much higher than would be normally found in buildings. Few cases of COVID-19 transmission have thus far been reported.^[11,136]^ HEPA filters make travelling in aircrafts less risky compared with other forms of transport with poorer ventilation properties. Nevertheless, transmission events have occurred on long-haul flights ^[137]^ and therefore it is advisable to continue with good behavioural standards during flights.^[11]^

One strategy that is often employed during periods of travel (including tournaments) to reduce the risk of infection is cohorting,^[138]^ which in effect creates small ‘bubbles’, beyond which an infection cannot proceed.^[129]^ In the context of travel, this might involve breaking the travelling party into smaller sub-groups (cohorts), so that each person interacts with only a few others.^[129]^ The formation of ‘travel bubbles’ greatly reduces connectivity of the whole travel party, thus reducing the risk of SARS-CoV-2 exposure, and an individual from contracting COVID-19, thus inhibiting the spread of the disease, and minimising the number of contacts identified who require isolation. This useful strategy may be expanded to camps and tournaments, where long stays are required in hotels and other types of accommodation. In this situation, enhanced protocols are required for the duration of the ‘travel bubble’ to prevent SARS-CoV-2 spreading within the group. This could also be extended to cover seating arrangements at meals, etc. Additionally, the ‘travel bubble’ should limit the interaction with others from outside of this cohort (e.g. waiters), and have strict entry criteria should the cohort need to be expanded (e.g. new players or an additional service added). Given the 14-day SARS-CoV-2 virus cycle, groups of athletes (and associated staff) should ensure that they follow a quarantine period for this duration of time, prior to becoming a ‘true bubble’, whereby physical distancing is not required, due to the cohort being confirmed as not infectious or infected with SARS-CoV-2. Within this bubble, no interaction should take place with individuals from outside of this group (e.g. family or friends) to ensure that the virus cannot enter the group.

### Transmission risk from spectators and media to sports staff and athletes

Risks from spectators, media and event staff is best understood by considering an event as a gathering comprising all present. Where physical distancing is breached, outdoors or indoors, four risk factors have been identified. These are, the size of gathering, the density (at the macro-level, this is a measure for the whole gathering [number of people in a given space], at the micro level this can be interpreted as proximity or distance between individuals), the duration (both overall time at the gathering, or time spent in any particular interaction as part of the gathering), and the extent of circulation within the gathering.^[130]^ This can be concurrently managed to mitigate each other in relation to community prevalence. In addition to the increased indoor transmission risk, an ‘indoor crowding effect’ has also been observed, where people naturally gather closer together.^[130,139,140]^ SARS-CoV-2 diagnostic testing of media or other personnel prior to athlete interacts (e.g. interviews) can be also used to reduce the SARS-CoV-2 transmission risk.

SARS-CoV-2 transmission risk for an event can be calculated based on community prevalence, event attendance, and the theoretical assumption that 50% of infected individuals are asymptomatic and presymptomatic. Consequently, if community infection is 4 in 1 000, 2 are aware they are infectious (e.g. symptomatic) and therefore would not attend, for a gathering of 10 000 people, 20 infectious individuals may be unknowingly present within the crowd. This risk can by reduced via an increase in mass asymptomatic testing of the community.

If the four identified mitigatable risks (size of gathering, density, duration, circulation) are applied to each fixture, the risk can be understood.^[130]^ For example, a Premiership soccer match is a large gathering, but with allocated seats, meaning the density is known, and the potential duration and circulation between the spectators and the group of athletes can be managed. At a ‘Sunday League’ fixture, despite being a smaller gathering, the SARS-CoV-2 transmission risk to the group of athletes may be greater, given that spectators are typically not in allocated seats, can circulate freely, and have the potential to become within close proximity of athletes. Some events (e.g. snooker, swimming) traditionally involve athletes being within closer proximity to spectators due to venue characteristics, which are also indoors.

## Overall system to mitigate SARS-CoV-2 transmission within sport

This review presents a summary of end-to-end SARS-CoV-2 transmission risk considerations for sport, providing an overview of transmission mechanisms and risks within specific scenarios and situations that are common for sport. Sports should aim to identify and reduce the chance of infectious individuals entering the environment, and then quantify and mitigate higher risk situations to prevent the spread of COVID-19 should an infectious individual enter the environment. The likelihood of this occurring increases during periods of high community COVID-19 prevalence, given the significant association observed between new weekly cases of COVID-19 in the community and professional rugby.^[80]^

Despite intensive monitoring and mitigation strategies, infectious individuals may still inadvertently enter the sporting environment. The identification and isolation of players and staff in elite sport should be undertaken with appropriate precision to prevent infected individuals remaining in the environment (e.g. resulting in potential virus transmission), and preventing low-risk contacts having to isolate, which potentially increases the risk of injury when returning. In addition, isolation may result in psychological strain, as well as causing wider disruption to competitions. Therefore, sports are required to identify individuals who have potentially been exposed to the virus and are subsequently required to isolate. This can be a challenging if national government guidelines have not been developed for sports.

In addition to COVID-19’s specific protocols, the availability and sharing of video footage or human-to-human proximity data and subject interview, allows accurate close contact identification once a SARS-CoV-2 positive case is found. Sport-specific contact tracing frameworks have been previously proposed (e.g. Team Sport Risk Exposure Framework (TS-REF),^[7]^ which has been used to identify increased risk sporting activities and to identify and isolate increased risk contacts during sporting activities.^[10]^

**Fig. 3:**
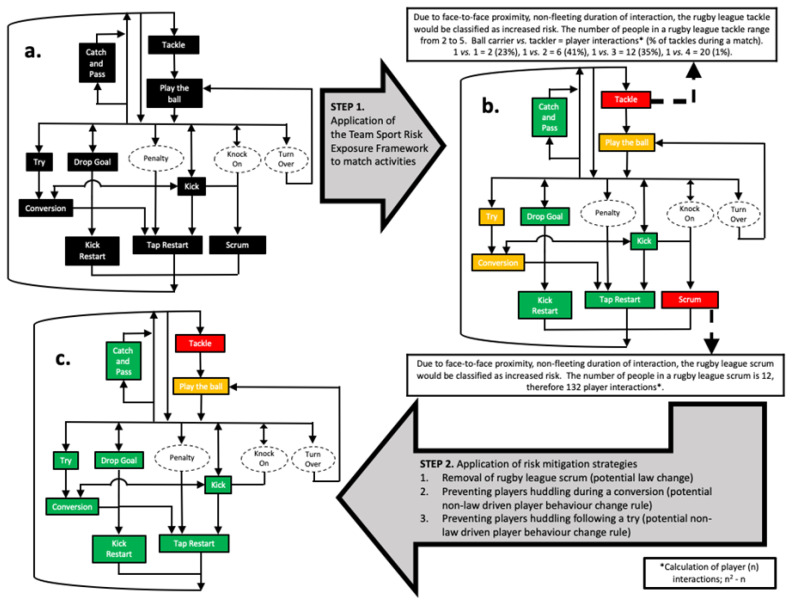
**(a)** A list of rugby league game-specific actions, **(b)** Application of Team Sport Risk Exposure Framework (TS-REF)^[7]^, **(c)** Rugby league game-specific actions following potential risk-reduction interventions

The TS-REF has been applied to rugby league match activities (Fig. 3a), which were consequently assigned a rating of ‘increased, medium or low risk’. This identified tackles and scrums as increased risk activities (Fig. 3b). Rule and player behaviour interventions were then developed and implemented to reduce the relative risk of rugby league match play from a SARS-CoV-2 transmission perspective (Fig. 3c). As a consequence, rugby league in England temporarily removed scrums during the COVID-19 pandemic to reduce the potential SARS-CoV-2 transmission risk. This also reduced the number of players that would have been required to isolate due to their involvement in increased risk activities, should an infectious player inadvertently participate in a match.

**Fig. 4:**
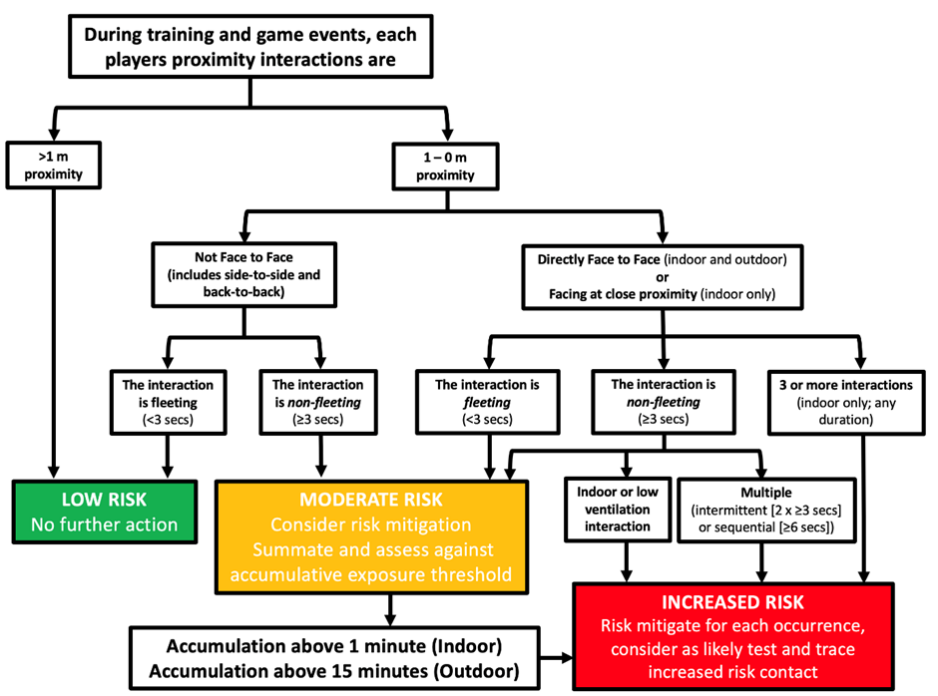
The Team Sport Risk Exposure Framework 2; TS-REF-2 to identify increased risk contacts in sport ^[88]^

The TS-REF has more recently been updated (The Team Sport Risk Exposure Framework 2; TS-REF-2, Fig. 4) to address the increased SARS-CoV-2 transmission risks indoors compared with outdoors. Guidance on practically determining indoor and outdoor environments has been proposed, which considers if the space has a roof, the air velocity at low levels, the volume of the space and density of people in the area, environmental conditions and CO_2_ concentration.^[88]^ In addition to government contact tracing guidelines, the sport-specific frameworks may further support the identification of close contacts within a sporting environment, helping to reduce the risk of the infectious individual staying in the environment.

## Conclusion

This review provides a summary of end-to-end SARS-CoV-2 transmission risk factors for sport and an overview of transmission mechanisms to be considered by all stakeholders. The risk of SARS-CoV-2 transmission is greatest indoors, and primarily influenced by the ventilation of the environment and (close) proximity of the individuals. The SARS-CoV-2 transmission risk outdoors, via water and from fomites, appears less than initially thought. Mitigation strategies include comprehensive end-to-end scenario planning of activities to optimise physical distancing, mask wearing and hygiene practice (for individuals, environment and equipment). The identification and removal of infectious individuals and their close contacts should be undertaken with appropriate precision to prevent further transmission. Sports should aim to undertake activities outdoors where possible, given the lower SARS-CoV-2 transmission risk, in comparison to indoor environments. Finally, the risk mitigation strategies presented may be applicable beyond the COVID-19 pandemic to reduce the risk of virus transmission in sport.

## Figures and Tables

**Fig. 1:**
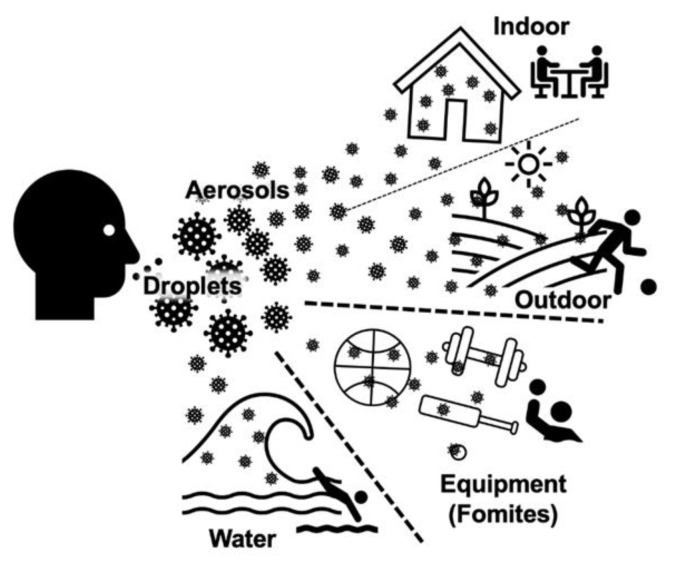
SARS-CoV-2 main transmission routes and risks for sport

**Table 1:** Recommended Personal Protective Equipment (PPE) guidance for specific clinical situations that may be encountered in the sporting environment ^[84,85]^

Medical and Professional Care Player Interaction	PPE Level
Maintaining physical distancing as advised; **NO** face-to-face contact risk	1
**NOT** maintaining 2 m distance; **WITH** face-to-face contact risk	2
Wound care, excluding oral/dental/nasal injuries	2
Uncomplicated concussion evaluation e.g. head injury assessment	2
Managing complex injuries, with no C-spine involvement e.g. isolated limb or joint injury	2
Medical emergency **WITHOUT** potential for airway compromise	2
Cardiac arrest* **WITHOUT** airway interventions therefore **WITH** face covered; Includes continuous compressions and automated external defibrillator use	2
Performing a nasopharyngeal swab	2
Nasal and oral procedures, e.g. epistaxis and oral injuries	3
Aerosol generating procedures	3
Medical emergency **WITH** potential for airway compromise e.g. complicated head injury, choking**	3
Cardiac arrest* – **WITH** airway intervention, therefore **WITHOUT** covered compressions	3

* Cardiac arrest scenarios have both options of Level 2 and Level 3 PPE to accommodate for availability in different situations.

** In cases of suspected choking, although Level 3 PPE provides the most appropriate protection, it is appreciated that an immediate life-saving intervention may be needed which may preclude donning of the extra garments. In these cases, Level 2 protection should be a minimum.

**Table 2:** Interventions with the potential to be aerosol generating procedures

Activity
Cardiopulmonary resuscitation (CPR) Airway management: any suction of upper airway, use of airway adjuncts and emergency surgical airway procedures Breathing management: any form of manual ventilation; bag-valve-mask ventilation using a viral filter is ideal, while mouth-to-mouth ventilation is not recommended Medical emergencies with altered levels of consciousness and a risk of comprising of the airways are potentially aerosol-generating procedures Nose and throat procedures, such as managing nasal epistaxis or oral lacerations

Note: Nebulising, high flow oxygen administration via facemask, nasopharyngeal swabbing and defibrillation are not considered aerosol generating procedures.

**Table 3:** COVID-19 R-numbers for a 1 400 m^3^ open-place office, occupied by 40 people for 8 hours each day, with a single pre/asymptomatic person present throughout the entire period ^[124,125]^

Scenario	Quanta production rate (quanta/hr)	Ventilation rate (4 L/s per person)	Ventilation rate (10 L/s per person)	Ventilation rate (20 L/s per person)
Quiet desk work (low viral shedder)	0.3	0.25	0.13	0.07
Quiet desk work (standard viral shedder)	1.0	0.84	0.42	0.24
Talking sedentary	5.0	4.00	2.10	1.20
Super-spreader (low)	20.0	14.00	7.60	4.40
Super-spreader (high)	100.0	35.00	26.00	18.0

The values presented in columns 3–5 above are the predicted R-values (i.e. the expected number of secondary COVID-19 infections arising from one infected person attending the office for 5 working days) for the specified ventilation rates. The quanta production rate associated with each activity scenario (column 1) is specified in column 2.
